# Occlusal Surface Changes in Single-Unit Versus Three-Unit Implant-Supported Zirconia Restoration: A Retrospective Observational Study

**DOI:** 10.7759/cureus.117202

**Published:** 2026-09-30

**Authors:** Jay Gohil, Shilpa Chawan, Mukesh Singla, Sanpreet S Sachdev, Goudu Lekha Pavani, Grishmi Niswade

**Affiliations:** 1 Department of Prosthodontics, K. M. Shah Dental College and Hospital, Sumandeep Vidyapeeth (Deemed to be University), Vadodara, IND; 2 Department of Periodontics, Tirumala Institute of Dental Sciences and Research Center, Nizamabad, IND; 3 Department of Orthodontics, Dasmesh Institute of Research and Dental Sciences, Faridkot, IND; 4 Department of Oral Pathology and Microbiology, Bharati Vidyapeeth (Deemed to be University) Dental College and Hospital, Mumbai, IND; 5 Department of Orthodontics and Dentofacial Orthopedics, Lenora Institute of Dental Sciences, Rajahmundry, IND; 6 Department of Periodontology, Swargiya Dadasaheb Kalmegh Smruti Dental College and Hospital, Nagpur, IND

**Keywords:** antagonist enamel wear, implant-supported zirconia, occlusal wear, single-unit crown, three-unit fixed dental prosthesis

## Abstract

Introduction: Implant-supported zirconia restorations (ISZRs) are increasingly used for posterior rehabilitation; however, differences in occlusal wear and associated clinical outcomes between single-unit restorations and three-unit fixed restorations remain clinically relevant. This study aimed to compare occlusal surface wear between single-unit implant-supported zirconia restorations (single-ISZRs) and three-unit implant-supported zirconia restorations (three-ISZRs) and evaluate antagonist enamel wear, occlusal adjustment requirements, surface roughness, and restoration-related complications.

Materials and methods: This retrospective observational study included 120 records, comprising 64 single-ISZRs and 56 three-ISZRs. Digital records at restoration delivery and at 6 and 12 months were evaluated. Occlusal surface wear and antagonist enamel wear were quantified using digital scan superimposition. Occlusal adjustments, surface roughness, and complications were assessed from clinical records. Continuous variables were analyzed using independent-samples t-tests with Welch’s correction where appropriate, while categorical variables were analyzed using Pearson's chi-square or Fisher's exact test. Statistical significance was set at p < 0.05.

Results: Baseline characteristics were comparable between the groups. At baseline, occlusal wear was 2.1 ± 0.8 μm for single-ISZR and 2.3 ± 0.9 μm for three-ISZR (p = 0.204). At 6 months, wear was 8.4 ± 2.1 and 11.2 ± 2.8 μm, respectively (p < 0.001), increasing at 12 months to 14.6 ± 3.4 and 19.8 ± 4.1 μm (p < 0.001). The baseline-to-12-month change was 12.5 ± 3.2 versus 17.5 ± 3.8 μm (p < 0.001). Antagonist enamel wear was also lower with single-ISZR (15.2 ± 3.8 vs. 19.7 ± 4.5 μm; p < 0.001). The overall complication rate was lower for single-ISZR than for three-ISZR (9.4% vs. 25.0%; p = 0.022).

Conclusion: Single-ISZRs demonstrated less occlusal and antagonist enamel wear and fewer overall complications than three-ISZRs during 12 months of follow-up.

## Introduction

Implant-supported zirconia restorations (ISZRs) are widely used for the rehabilitation of posterior edentulous spaces because of their favorable mechanical properties, esthetics, biocompatibility, and wear resistance [1]. In particular, monolithic zirconia has gained popularity because it eliminates the veneering layer that may be susceptible to chipping and fracture [2]. However, the long-term behavior of zirconia restorations under functional loading remains clinically relevant, particularly with respect to occlusal surface changes and their potential influence on opposing dentition [3]. Occlusal wear is a progressive process that may alter the morphology and vertical dimension of the restoration, while excessive wear of the antagonist enamel may compromise the natural tooth structure [4]. The magnitude of these changes may also be influenced by the restoration design, occlusal contacts, location within the dental arch, opposing dentition, and distribution of functional forces.

Single-unit implant-supported zirconia restorations (single-ISZRs) and multi-unit fixed dental prostheses differ in their structural configurations and occlusal force distribution [5]. In three-unit implant-supported zirconia restorations (three-ISZRs), occlusal loading is transferred across multiple units and implant-supported components, potentially resulting in a different pattern of functional surface changes compared to an individual implant-supported crown [6]. Clinical monitoring of these changes is therefore important for determining whether restoration configuration influences the preservation of occlusal morphology and opposing enamel.

Conventional clinical assessment of wear may be subjective and may not adequately quantify small changes in the restorative surfaces. Digital intraoral scanning and three-dimensional superimposition provide a non-invasive approach for detecting and quantifying subtle changes in occlusal morphology over time [7]. In the present study, digital scan datasets obtained at delivery and during follow-up were used to assess the vertical occlusal surface height loss in ISZRs. The study also considered associated clinical outcomes, including antagonist enamel wear, the requirement for occlusal adjustment, occlusal surface roughness, and restoration-related complications.

The primary objective of this study was to compare the longitudinal occlusal surface wear between single-ISZR and three-ISZR. The secondary objective was to compare antagonist enamel wear, frequency of occlusal adjustments, occlusal surface roughness, and restoration-related complications between the two restoration types during follow-up.

## Materials and methods

This retrospective observational cohort study was conducted in the Department of Prosthodontics, K. M. Shah Dental College and Hospital, Sumandeep Vidyapeeth (Deemed to be University), Vadodara, India, in 2022. This study involved a retrospective review of the clinical records of patients who had received ISZRs during the preceding three years, from January 2019 to December 2021. Institutional records were screened systematically, and eligible records containing complete clinical documentation and digital intraoral scan datasets at restoration delivery, 6-month follow-up, and 12-month follow-up were included. The study protocol was reviewed and approved by the Institutional Ethics Committee of the college (SVIEC/Dent/2022/Feb/035). Considering the retrospective nature of the investigation, the absence of any additional intervention, and the use of previously recorded clinical data, the Institutional Ethics Committee waived the requirement for informed consent. All patient information was anonymized and coded before the analysis, and the study was conducted in accordance with the principles of the Declaration of Helsinki.

Because the study used a retrospective record-based design, all available records that met the predefined eligibility criteria during the study period were considered for inclusion. A total of 120 eligible records were identified, comprising 64 single-ISZRs and 56 three-ISZRs. This census-based approach minimized the selection of records based on outcome availability.

The study population consisted of patients who had received either a single-ISZR or a three-ISZR in the posterior region, involving premolar or molar sites. Patients were included when complete demographic information, treatment records, implant-related documentation, restoration details, and digital intraoral scan files were available at baseline and during the follow-up. A minimum follow-up period of 12 months after definitive restoration delivery was required. Records were excluded when there was a documented history of bruxism or significant parafunctional activity, excessive occlusal adjustment beyond the predefined routine clinical protocol, incomplete digital scan or clinical records, or uncontrolled systemic disease that could substantially influence bone or metabolic status. Restorations that experienced fracture or debonding during follow-up were retained in the study and included in the complication analysis.

All implant placement procedures were performed by an experienced prosthodontist (JG) using the NobelActive implant system (Nobel Biocare Services AG, Kloten, Switzerland). Nobel Biocare-manufactured Nobel Active implants were selected because of their high primary stability and suitability for posterior implant-supported rehabilitation. Implant osteotomy preparation and placement were performed according to the manufacturer's recommended surgical protocol, with implant diameter and length selected according to the available bone dimensions and prosthetic requirements. Following osseointegration and the completion of the restorative phase, definitive implant-supported restorations were fabricated. The implant-supported restorations consisted of single-ISZR and three-ISZR. The zirconia restorations were fabricated using the Nobel Procera restorative system (Nobel Biocare AB, Göteborg, Sweden), with full-contour zirconia selected for definitive restorations.

Definitive restorations were designed digitally using the 3Shape Dental System 2019 (3Shape A/S, Copenhagen, Denmark). The restorations were designed to reproduce the patient's existing occlusal anatomy, while maintaining appropriate proximal contacts, occlusal contacts, emergence profiles, and implant-prosthetic dimensions. Following digital design, zirconia restorations were manufactured according to the manufacturer's recommended CAD/CAM protocol, sintered, and finished before clinical delivery. Definitive restorations were evaluated intraorally for marginal adaptation, proximal contact, occlusal contact, and passive seating. Occlusal adjustments, when clinically required, were performed using fine-grit diamond instruments followed by polishing according to the manufacturer's recommended protocol. The cementation protocol and the type of luting agent were obtained from clinical records.

Digital impressions were obtained using a 3Shape TRIOS 3 intraoral scanner (3Shape A/S, Copenhagen, Denmark). The intraoral scanner was calibrated according to the manufacturer's recommendations prior to scanning. For each eligible restoration, the maxillary or mandibular arch containing the implant-supported restoration and the opposing arch were scanned along with the occlusal relationship. Scanning was performed using a standardized full-arch scanning sequence to ensure adequate capture of the restorative surface, adjacent teeth, and opposing dentition. Digital datasets were stored in standard tessellation language (STL) format. Digital scans were retrieved at restoration delivery and 6 and 12 months after definitive restoration placement (Figure 1). The baseline scan obtained at delivery was considered the reference dataset, whereas the subsequent scans were considered follow-up datasets. Baseline scans obtained at restoration delivery were designated as the reference datasets. Occlusal wear was calculated from the change in vertical occlusal surface height between the baseline reference scan and each follow-up scan at 6 and 12 months. Thus, baseline-to-baseline change was considered zero, and any small surface deviation observed during baseline registration was regarded as measurement variability rather than biological wear.

**Figure 1:**
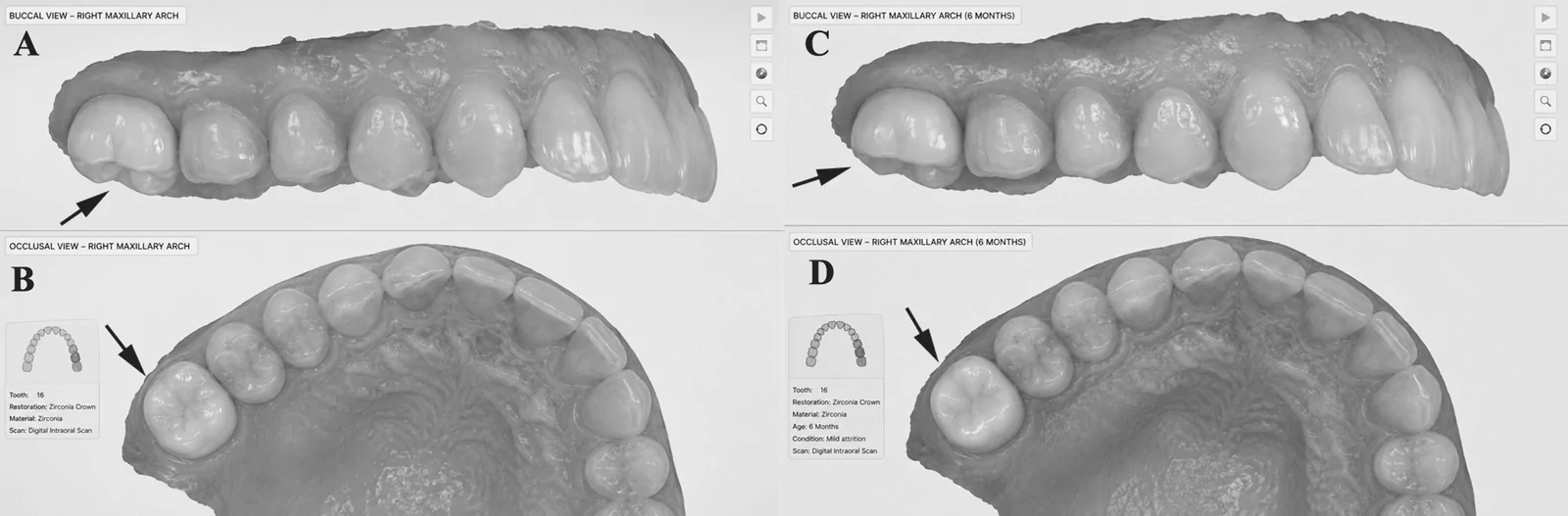
Representative digital intraoral scans demonstrating the assessment of the implant-supported zirconia restoration at baseline and 6 months. Single-unit implant-supported zirconia restoration (black arrow) of the right maxillary arch at baseline (A) buccal (B) occlusal view, and at 6-month follow-up (C) buccal (D) occlusal view. Image created using Geomagic Control X 2018.0 (3D Systems, Rock Hill, South Carolina, USA).

The STL datasets were imported into Geomagic Control X 2018.0 (3D Systems, Rock Hill, South Carolina, USA) for three-dimensional surface comparison and quantitative assessment of occlusal changes. Geomagic Control X is a metrology-grade software platform used for three-dimensional inspection, surface comparison, alignment, and measurement of digital meshes. The baseline and follow-up datasets were initially aligned using the initial alignment function, followed by best-fit surface alignment. Stable non-wear areas of the restoration and adjacent anatomical structures were used as reference regions to minimize registration errors.

Vertical occlusal surface change was assessed by superimposing the baseline and follow-up digital images using stable anatomical reference structures. A standardized horizontal reference plane was established, and the perpendicular distance from this reference plane to the corresponding occlusal surface was measured at baseline and follow-up. The change in vertical occlusal position was calculated as the difference between the follow-up and baseline measurements, with the absolute value representing the magnitude of occlusal surface change. The primary outcome was vertical occlusal surface height loss expressed in micrometers (μm) (Figure 2). Measurements were obtained at baseline and 6 and 12 months, and the change from baseline to 12 months was calculated for each restoration. Surface deviation maps were generated to visualize the areas of material loss and quantify the magnitude of occlusal alteration.

**Figure 2:**
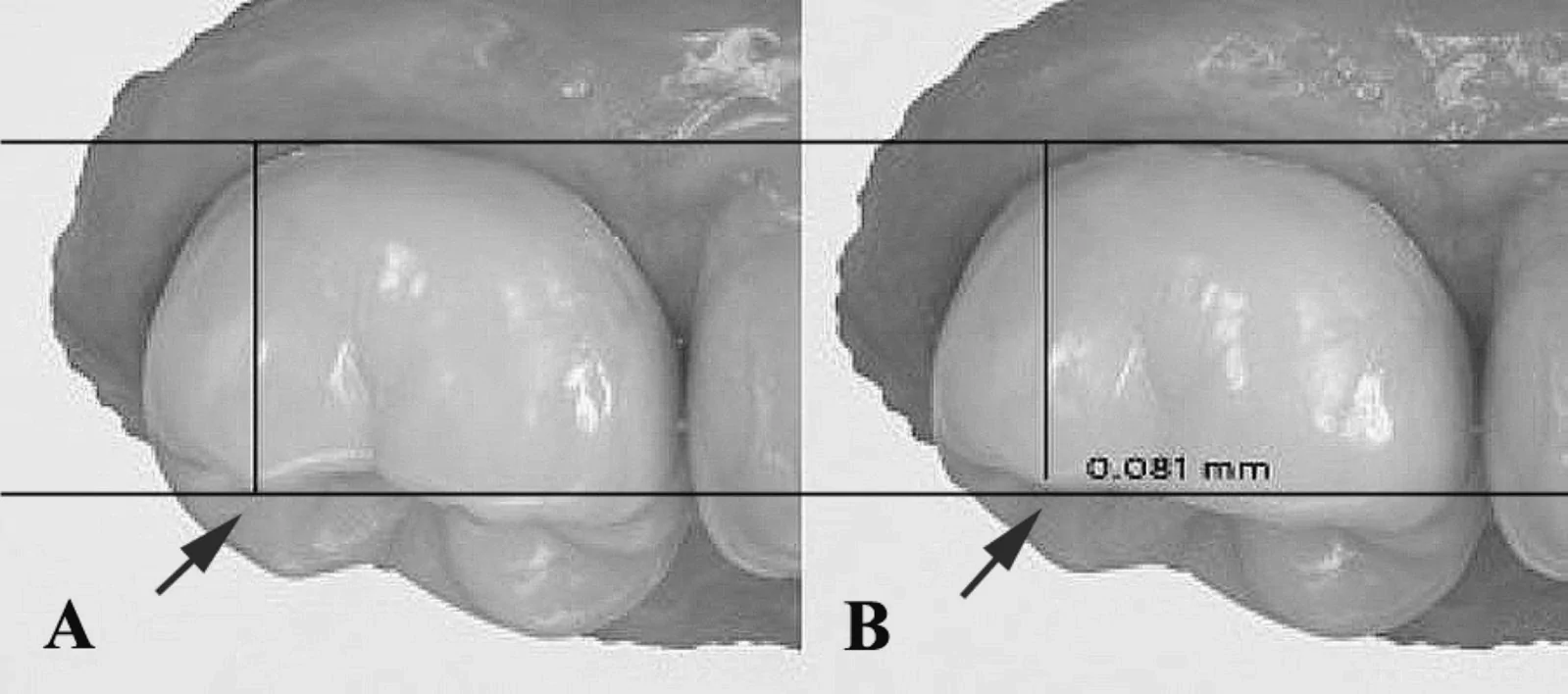
Representative three-dimensional digital scan measurement of occlusal surface loss at the distolingual cuspal tip. Superimposed digital images of an implant-supported zirconia restoration demonstrating the measurement of vertical occlusal surface change between baseline (A) and follow-up (B), with a representative surface deviation of 0.081 mm (black arrow). 0.081 mm = 81 μm. Image created using Geomagic Control X 2018.0 (3D Systems, Rock Hill, South Carolina, USA).

Antagonist dentition was evaluated using the corresponding digital scan datasets. Vertical dimensional changes in the opposing natural tooth surfaces were measured in micrometers using the same three-dimensional superimposition procedure. The opposite tooth surface was isolated and compared between the baseline and 12-month datasets after appropriate registration. Antagonist enamel wear was recorded as the secondary outcome. In addition, clinical records were reviewed for the requirement of occlusal adjustment at restoration delivery, 6-month recall, and 12-month recall. Occlusal surface condition at the 12-month evaluation was categorized as smooth or rough/surface degradation. Restoration-related complications were recorded throughout the follow-up period and included framework or veneer chipping, framework fracture, restoration debonding, and the overall complication rate.

Digital datasets were anonymized and assigned study-specific codes by an investigator who was not involved in the outcome assessment (JG). The coded datasets were then independently evaluated by two calibrated examiners (SC and SSS) from institutions independent of the study center. Both examiners were blinded to patient identity, treatment group, and clinical outcomes recorded during follow-up. Neither examiner participated in implant placement, prosthodontic treatment, nor in clinical follow-up of the included patients.

Before commencement of the main assessment, both examiners performed a standardized calibration exercise using a representative subset of records that were not included in the final analysis. Each examiner independently identified the restoration surface, selected reference regions, performed digital superimposition, and measured occlusal surface changes. The measurements obtained by the two examiners were compared to determine inter-examiner reliability. The same examiner repeated the measurements after a two-week interval to determine intra-examiner reliability. Reliability was assessed using the intraclass correlation coefficient (ICC) with a two-way mixed-effects model for absolute agreement. The intra-examiner ICC was 0.94 (95% CI, 0.91-0.96), whereas the inter-examiner ICC was 0.92 (95% CI, 0.88-0.95), indicating excellent reproducibility. The mean of two calibrated measurements was used in the final analysis.

To ensure allocation concealment during data analysis, all patient records were assigned a unique numerical identification code prior to statistical processing. Information identifying the patient, operator, restoration group, and original clinical records was removed from the analytical dataset. The coded dataset was subsequently provided to an independent statistician who was not involved in clinical treatment, record screening, or digital measurements (MS). The statistician was blinded to the identity of the patients and the examiner who performed each measurement.

Statistical analyses were performed using IBM SPSS Statistics for Windows, Version 25 (Released 2017; IBM Corp., Armonk, New York). Continuous variables, including age, follow-up duration, occlusal surface wear, and antagonist enamel wear, were assessed for distribution using the Shapiro-Wilk test. Normally distributed continuous variables were expressed as mean ± standard deviation and compared between the single-ISZR and three-ISZR groups using the independent-samples t-test, with Welch's correction applied when equality of variances was not assumed. Categorical variables were expressed as frequencies and percentages and were compared using Pearson's chi-square test. Fisher's exact test was used when one or more expected cell frequencies were less than 5. Effect sizes are reported as Cohen's d for continuous variables, phi (φ) for categorical comparisons, and odds ratios (ORs) for binary complication outcomes. Statistical significance was defined as a two-sided p-value < 0.05.

## Results

A total of 120 records were analyzed, including 64 single-ISZRs and 56 three-ISZRs. Baseline characteristics were comparable between the groups, with no significant differences in age, sex, smoking status, arch location, region, or follow-up duration (Table 1).

**Table 1:** Baseline demographic and clinical characteristics of the study groups. Data are presented as mean ± standard deviation (SD) for continuous variables and frequency (percentage) for categorical variables. p-values were obtained using the independent-samples t-test for continuous variables, with Cohen's d as the effect size, and Pearson's chi-square test for categorical variables, with the phi coefficient as the effect size. ISZR: Implant-supported zirconia restoration; df: degrees of freedom.

Characteristic	Overall (N = 120)	Single-ISZR (n = 64)	Three-ISZR (n = 56)	Test statistic (df)	p-value	Effect size
Age (years), Mean ± SD	59.2 ± 9.0	58.4 ± 9.2	60.1 ± 8.7	t = -1.04 (df = 118)	0.301	d = -0.19
Sex, n (%)						
Male	64 (53.3%)	34 (53.1%)	30 (53.6%)	χ² = 0.002 (df = 1)	0.961	φ = 0.004
Female	56 (46.7%)	30 (46.9%)	26 (46.4%)			
Smoking status, n (%)						
Non-smoker	94 (78.3%)	50 (78.1%)	44 (78.6%)	χ² = 0.004 (df = 1)	0.953	φ = 0.005
Smoker	26 (21.7%)	14 (21.9%)	12 (21.4%)			
Arch location, n (%)						
Maxilla	71 (59.2%)	38 (59.4%)	33 (58.9%)	χ² = 0.002 (df = 1)	0.960	φ = 0.005
Mandible	49 (40.8%)	26 (40.6%)	23 (41.1%)			
Region, n (%)						
Premolar	52 (43.3%)	28 (43.8%)	24 (42.9%)	χ² = 0.010 (df = 1)	0.922	φ = 0.009
Molar	68 (56.7%)	36 (56.3%)	32 (57.1%)			
Follow-up duration (months), Mean ± SD	12.5 ± 1.1	12.4 ± 1.1	12.6 ± 1.0	t = -1.04 (df = 118)	0.302	d = -0.19

Occlusal surface wear was similar at baseline but was significantly greater in the three-ISZR group at 6 and 12 months (Table 2). At 12 months, wear was 14.6 ± 3.4 μm for single-ISZR and 19.8 ± 4.1 μm for three-ISZR (p < 0.001). The baseline-to-12-month increase was 12.5 ± 3.2 and 17.5 ± 3.8 μm, respectively (p < 0.001).

**Table 2:** Comparison of occlusal surface wear between single-ISZR and three-ISZR. *Significance at p < 0.05. Data are presented as mean ± standard deviation in micrometers (μm). p-values were obtained using the independent-samples t-test; Cohen's d represents the standardized effect size. ISZR: Implant-supported zirconia restoration; CI: confidence interval; df: degrees of freedom.

Time point	Single- ISZR (n = 64) μm	Three-ISZR (n = 56) μm	Mean difference (95% CI)	t value (df)	p-value	Cohen's d
Baseline (delivery)	2.1 ± 0.8	2.3 ± 0.9	−0.2 (−0.5, 0.1)	−1.29 (118)	0.204	−0.24
6-month follow-up	8.4 ± 2.1	11.2 ± 2.8	−2.8 (−3.7, −1.9)	−6.24 (118)	< 0.001*	−1.14
12-month follow-up	14.6 ± 3.4	19.8 ± 4.1	−5.2 (−6.6, −3.8)	−7.59 (118)	< 0.001*	−1.39
Change (Δ) baseline→12-month	12.5 ± 3.2	17.5 ± 3.8	−5.0 (−6.3, −3.7)	−7.82 (118)	< 0.001*	−1.43

Antagonist enamel wear at 12 months was also significantly lower with single-ISZR than with three-ISZR (15.2 ± 3.8 vs. 19.7 ± 4.5 μm, p < 0.001). Occlusal adjustments were more frequent in the three-ISZR group at delivery, 6 months, and 12 months, although these differences were not statistically significant. Occlusal roughness was likewise more frequent with three-ISZR (p = 0.091). Individual complications, including chipping, fracture, and debonding, were more frequent in the three-ISZR group but did not differ significantly between the groups. However, the overall complication rate was significantly higher for three-ISZRs than for single-ISZRs (p = 0.022) (Table 3).

**Table 3:** Comparison of secondary clinical outcomes and restoration-related complications between the study groups. *Significance at p < 0.05. Data are presented as frequencies (percentages) for categorical variables and mean ± standard deviation (SD) for continuous variables; p-values were obtained using Pearson's chi-square test with the phi coefficient as the effect size, or Fisher's exact test for categorical variables, and the independent-samples t-test for continuous variables. ISZR: Implant-supported zirconia restoration; df: degrees of freedom.

Outcome variable	Single-ISZR (n = 64)	Three-ISZR (n = 56)	Test statistic (df)	p-value	Effect size
Antagonist enamel wear (μm)					
At 12 months, Mean ± SD	15.2 ± 3.8	19.7 ± 4.5	t = −5.87 (df = 118)	< 0.001*	d = −1.09
Need for occlusal adjustment, n (%)					
At delivery	18 (28.1%)	22 (39.3%)	χ² = 1.67 (df = 1)	0.196	φ = 0.12
At 6-month recall	10 (15.6%)	14 (25.0%)	χ² = 1.64 (df = 1)	0.200	φ = 0.12
At 12-month recall	6 (9.4%)	12 (21.4%)	χ² = 3.40 (df = 1)	0.065	φ = 0.17
Occlusal roughness (Qualitative), n (%)					
Smooth	52 (81.3%)	38 (67.9%)	χ² = 2.86 (df = 1)	0.091	φ = 0.15
Rough/surface degradation	12 (18.8%)	18 (32.1%)			
Complications, n (%)					
Framework/veneer chipping	3 (4.7%)	7 (12.5%)	Fisher's exact	0.186	OR = 0.34
Framework fracture	1 (1.6%)	3 (5.4%)	Fisher's exact	0.338	OR = 0.28
Restoration debonding	2 (3.1%)	4 (7.1%)	Fisher's exact	0.416	OR = 0.42
Total complication rate	6 (9.4%)	14 (25.0%)	χ² = 5.25 (df = 1)	0.022*	φ = 0.21

## Discussion

The present retrospective study demonstrated that single-ISZRs exhibited significantly less occlusal surface wear than three-ISZRs over 12 months. Although the groups showed comparable wear at baseline, the difference became significant at 6 months and increased further at 12 months. This finding suggests that restoration configuration may influence the distribution and concentration of functional occlusal forces [8]. The greater occlusal surface change observed in the three-ISZR group may be related to differences in prosthetic configuration and load transfer, as previous three-dimensional finite-element analysis has demonstrated that prosthesis and implant design can substantially influence stress distribution within implant-supported fixed partial dentures [9]. Differences in connector geometry, rigidity of the prosthetic framework, occlusal contact distribution, and localized stress concentration may therefore contribute to the greater surface alteration observed in the three-ISZR group [10]. The large effect sizes observed at 6 and 12 months further indicate that this was not merely a statistically significant difference, but also a potentially meaningful difference in restorative wear.

The progressive increase in wear in both groups is consistent with the expected behavior of zirconia restorations subjected to repeated masticatory loading [3]. However, the substantially greater change in the three-ISZR group may reflect the cumulative effect of repeated functional contacts over a larger occlusal restorative surface. Previous clinical studies have demonstrated that monolithic zirconia undergoes measurable wear during function, although the magnitude varies considerably according to surface finishing, occlusal conditions, measurement methodology, and observation duration [11,12]. A systematic review reported progressive antagonist wear associated with monolithic zirconia over time, supporting the present observation of increasing occlusal changes during follow-up [13]. The use of digital three-dimensional superimposition in the present study may also have enabled the detection of relatively small changes that are difficult to identify clinically.

A clinically important finding was the significantly greater antagonist enamel wear associated with the three-ISZRs. At 12 months, antagonist wear was approximately 30% higher in the three-ISZR group than in the single-ISZR group. This finding is biologically plausible because increased restorative surface alteration and potentially different patterns of occlusal contact may increase the cumulative interaction between zirconia and opposing dentition. Previous clinical studies have confirmed that monolithic zirconia can produce measurable antagonist enamel wear, although generally within clinically acceptable ranges [11-13]. Importantly, the literature is inconsistent. A randomized clinical study reported that polished monolithic zirconia did not produce accelerated enamel wear compared with metal-ceramic restorations or natural enamel [14]. This apparent contrast may be explained by the differences in surface polishing, occlusal adjustment, restoration design, antagonist characteristics, follow-up duration, and wear measurement techniques. A systematic review similarly emphasized the importance of surface texture, with polished zirconia producing less antagonist wear than glazed surfaces [15].

The requirement for occlusal adjustment was consistently higher in the three-ISZR group at delivery and during follow-up, although the differences were not statistically significant. This trend may reflect biomechanical differences between implant-supported and natural dentitions. Unlike natural teeth, which exhibit physiological mobility through the periodontal ligament, osseointegrated implants have markedly limited mobility and lack periodontal ligament-mediated proprioception, which may influence the establishment and maintenance of occlusal contacts [16]. Similarly, surface roughness was more frequently observed in three-ISZRs, although the difference was not statistically significant. Previous evidence indicates that zirconia surface characteristics, particularly surface roughness and finishing, can influence its interaction with antagonist enamel; therefore, appropriate polishing following occlusal adjustment may help minimize unfavorable surface effects [12,15].

The overall complication rate was significantly higher in the three-ISZR group, although individual complications, such as chipping, fracture, and debonding, did not differ significantly. This pattern suggests that the significant difference was driven by the cumulative occurrence of several relatively infrequent complications, rather than by one specific failure mechanism. The higher complication rate observed with three-ISZRs may be related to the greater mechanical complexity of multi-unit restorations, including connector regions and multiple implant-prosthetic interfaces, which may provide additional sites susceptible to mechanical complications [17]. Clinically, this finding supports careful prosthetic design, adequate connector dimensions, accurate occlusal adjustment, and regular maintenance of multi-unit implant restorations.

The clinical implications of these findings should be interpreted based on the clinical indications of the respective restoration design. Single-ISZRs and three-ISZRs are not necessarily alternative treatments for the same clinical situation. Rather, restoration selection is primarily determined by the number and distribution of missing teeth, implant availability, bone and soft-tissue conditions, and prosthetic requirements. Therefore, the present findings should not be interpreted as suggesting that single crowns should preferentially replace three-ISZRs. However, when either restoration is clinically indicated, the observed differences in wear and complications provide useful information for subsequent maintenance. For single-ISZR, the relatively lower occlusal and antagonist wear observed in the present study suggests a favorable short-term wear profile. For three-ISZRs, the greater occlusal and antagonist wear and higher overall complication rate highlight the importance of precise occlusal adjustment, appropriate surface finishing and polishing, and periodic evaluation of the restoration and opposing dentition. Thus, the findings are more relevant to postoperative monitoring and maintenance than to determining the indication for one restoration type.

This study had several limitations. The retrospective observational design limited control over potential confounding factors and precluded the establishment of causal relationships. The follow-up period was limited to approximately 12 months, which may not adequately reflect long-term zirconia wear or prosthetic survival. The single-center setting and relatively modest sample size may also limit the generalizability of the findings. Factors such as individual chewing behavior, occlusal force, surface-finishing protocols, antagonist morphology, and other prosthetic characteristics could not be completely standardized or controlled retrospectively. In addition, the study compared restoration configurations rather than different zirconia materials or surface treatments. The longitudinal wear assessment was based on repeated observations of the same restorations, and the primary between-group comparisons did not incorporate a formal repeated-measures model or group × time interaction. Therefore, the reported findings should be interpreted as associations rather than causal effects. Prospective multicenter studies with longer follow-up, standardized surface-finishing protocols, quantitative occlusal force assessment, and appropriate longitudinal statistical modeling are warranted.

## Conclusions

Within the limitations of this retrospective observational study, single-ISZR was associated with less occlusal surface wear and antagonist enamel wear than three-ISZRs over the 12-month follow-up period. Multi-unit restorations also demonstrated a higher overall complication rate. These findings indicate an association between restoration configuration and occlusal and mechanical outcomes and should not be interpreted as evidence of a causal effect. Careful occlusal adjustment, surface finishing, and appropriate long-term maintenance may be relevant considerations, particularly for three-ISZRs.
